# A “*Familiar Foe Revisited*”: examining the relationship between endemic Burkitt’s lymphoma and changing malaria admissions in the coastal region of Kenya

**DOI:** 10.1186/s12885-025-15450-9

**Published:** 2025-12-18

**Authors:** Michael K. Mwaniki, Shebe Mohammed, Nyambura Kariuki, Dalton C. Wamalwa, Fred Mutisya, Charles R. Newton

**Affiliations:** 1 Department of Pediatrics & Child Health, School of Medicine, University of Nairobi, Nairobi, Kenya; 2 Afya Research Africa (ARA), PO Box 20880-00202, Nairobi, Kenya; 3 Amref International University, Nairobi, Kenya; 4 Centre for Geographic Medicine Research Coast, Kenya Medical Research Institute, P.O. Box 230, Kilifi, Kenya; 5 Centre for Health AI Research, Innovation and Implementation (CHAIIRI), Nairobi, Kenya; 6 Department of Psychiatry, University of Oxford, Oxford, UK

**Keywords:** P. falciparum malaria, Burkitt’s lymphoma, Epstein‒Barr virus, BurkitL belt Burkitt’s lymphoma Belt, Incidence

## Abstract

**Background:**

Burkitt’s lymphoma (BL) is a type of non-Hodgkin lymphoma that may account for more than 40% of childhood malignancies in tropical Africa. The endemic version is common in equatorial Africa, where a BL belt has been mapped. The role of *P. falciparum malaria* infection in BL has been postulated but not substantiated. The decrease in *P. falciparum malaria* infection offers an opportunity to examine this association.

**Methods:**

In this study, we utilized data collected over three decades (1990–2020) and examined the trends in annual admission incidence rates of Burkitt’s lymphoma among pediatric admissions (0–14 years) in relation to the reduction in malaria admissions.

**Findings:**

Ninety-five patients with Burkitt’s lymphoma were identified, of whom 72 (75.8%) were male. During the first epoch (1990–1999) and second decade, 29 cases and 62 cases were diagnosed, resulting in 10-year cumulative incidence rates of 93.1 cases and 130.3 cases per 100,000, respectively. In the third decade, 2010–2020, there were only 4 cases (cumulative incidence of 10.2 cases per 100,000). With one-way ANOVA, the F statistic for within- and between-group comparisons was significant (*p* < 0.0001), indicating that the decline across the three epochs was statistically significant. Similarly, the median parasite density decreased from 13,966 (Q1:2090, Q3:126,000) in the first epoch to 7,224 (Q1:1566, Q3:109,200) in the third epoch (Kruskal‒Wallis chi-square test, 12.3; *P* = 0.0021). One-way ANOVA for within- and between-group comparisons was equally significant (*p* < 0.001). The correlation coefficient between endemic BL and *P. falciparum malaria* infection was 0.53, indicating a strong positive correlation (*P* = 0.0024), implying that as *P. falciparum malaria* infection decreased, the endemic BL incidence rate decreased.

**Interpretation:**

There has been a significant reduction in the annual incidence rates of Burkitt’s lymphoma in the coastal region of Kenya. It is plausible that this decrease can be explained by an equally sustained and significant decline in the number of falciparum malaria infections.

## Introduction

Cancer directly causes nearly 10 million deaths annually [1], with estimates suggesting that 85% of global childhood cancers occur in low- and middle-income countries (LMICs) [2]. Each year, nearly half a million children and adolescents are diagnosed with cancer [3]. In high-income countries, over 80% of childhood cancers are cured or go into remission [4]. However, in LMICs, mortality is extremely high, with a cure rate of less than 50% [4]. This high mortality is driven by a myriad of factors, including a lack of diagnostic infrastructure (under diagnosis and/or misdiagnosis), a lack of timely access to care with advanced presentations, treatment abandonment, death from toxicity and a continuous cycle of relapses due to a lack of optimized treatment [2, 5]. Furthermore, health information systems are still in their formative stages in LMICs; hence, available statistics on cancer burden are likely to be grossly underestimates.

Leukemia is a common childhood cancer worldwide [4]; however, in tropical Africa, non-Hodgkin lymphoma predominates, accounting for up to 40% of reported childhood malignancies [6]. More than 30 variants of non-Hodgkin’s lymphoma have been described [7]. Burkitt’s lymphoma (BL) is the most common lymphoma in children living in tropical Africa, especially East Africa [8]. BL is a B-cell non-Hodgkin lymphoma characterized by the translocation and deregulation of the *MYC* gene on the 8th chromosome [8]. Three varieties of this aggressive B-cell lymphoma have been described: endemic, sporadic, and immune deficiency -associated [8]. Overall, BL is endemic to areas where *P. falciparum malaria* is holo-endemic, especially tropical regions, and hence, the term endemic BL. Although histological examination reveals virtually no difference from other forms of BL, the endemic form frequently presents with tumors of the jaw in children, but other sites, including those with abdominal involvement, are present [9].

There are three postulated predisposing factors for endemic BL: infection with Epstein‒Barr virus (EBV), *Plasmodium falciparum infection*, and translocation, which usually involves chromosomes 8 and 14 [10, 11]. The incidence rates of endemic BL are many-fold higher in equatorial Africa, where two of the known predisposing factors (falciparum malaria and EBV infections) are common [8]. Given that EBV is ubiquitously distributed globally even in regions with very low incidence rates of BL, the control of falciparum malaria infection may prevent endemic Burkitt lymphoma [12]. This hypothesis has not been fully examined.

The coastal region of Kenya provides an ideal setting for studying the epidemiology of endemic BL, as it lies within the BL belt just south of the equator [9, 13]. Importantly, while falciparum malaria accounted for more than 40% of inpatient pediatric admissions for decades, a considerable decline was recorded from 2008 [14, 15]. Given the aforementioned studies reporting a decline in the incidence of *P. falciparum malaria*, we investigated the change in the epidemiology of endemic BL as well as whether there have been significant changes associated with the changing documented temporal trends for *P. falciparum malaria*.

### Methodology

Kilifi County Referral Hospital is set at sea level in a *P. falciparum malaria* endemic area and has a catchment population of approximately half a million people. The hospital equally lies within the endemic Burkitt’s lymphoma belt in Kenya. It has a general pediatric inpatient ward with 60 beds, a high dependency unit with six (6) beds and seven (7) cots and a newborn unit with 42 cots. Kilifi County Referral Hospital further hosts the Kenya Medical Research Institute (KEMRI) Centre for Geographic Medicine Research Coast and the KEMRI-Wellcome Trust Research Program. Since 1999, through the research program, continuous surveillance has been initiated at the hospital with the aims of (i) providing a sampling frame for long-term epidemiological and related studies; (ii) evaluating the impact of new community-based interventions against infectious diseases; and (iii) establishing a base for clinical trials and other advanced interventional studies. On admission, discharge or death, standardized clinical and laboratory data are collected for each child [14]. These include a complete history and physical examination, a routine complete blood count, a malaria slide for examination, and blood culture for surveillance of invasive bacterial infections. Other tests (lumbar puncture for cerebrospinal fluid analysis; urea, creatinine and electrolytes; urinalysis; and urine culture, imaging or radiological examination) are guided by clinical indications. Overall, the management of children admitted follows the Kenya National and World Health Organization’s recommendations [16].

Aside from the above routine investigation, for children with a preliminary clinical impression of “possible malignancy”, tissue specimens (excision biopsy, fine needle aspiration, bone marrow aspiration and cerebral spinal fluid analysis) are obtained for pathological examination and definitive diagnosis.

### Study population

This study utilized data from all children aged 0–14 years admitted to the study site from 1990 to 2020. From this dataset, total admissions aged 0–14 years and admissions with a final discharge diagnosis of Burkitt’s lymphoma (confirmed by tissue specimen examination) were identified. Likewise, children with a final diagnosis of *P. falciparum malaria* (confirmed by blood slide microscopic examination) were obtained. To control for changes in the population in the catchment area, the at-risk population (0–14 years old) was estimated from census data for Kilifi County and used as the denominator. To estimate this number, census estimates from 1989, 1999, 2009 and 2019 were utilized. This resulted in estimated populations at risk of 165,674, 231,157 and 310,615 for the 1st (1990–1999), 2nd (2000–2009) and 3rd (2010–2020) epochs, respectively. The annual growth rate of the population under 14 years steadily increased over the three decades at the following annual rates: from 1989 to 1999 (6,548.3 per year), 1999–2009 (7,945.8 per year), and from 2009 to 2019, it further increased to 9,630.0 per year. This growth rate was input into a piecewise linear interpolation calculation to smooth the population numbers. This approach assumed a linear growth rate with uniformity for each age group within the 0–14-year range.

### Sample size

To examine the minimum number of BL cases needed to answer the main objective (sample size), we applied the approach for a longitudinal study estimating the main effect of a time-varying exposure as described by *Basagaña and Spiegelman* [17]. In this case, an a priori assumption that *P. falciparum malaria* infection influences endemic Burkitt lymphoma was made; hence, malaria infection was taken as the time-varying exposure. Data for *P. falciparum malaria* point prevalence were obtained from a 25-year-long study performed from 1990 to 2014 among children admitted to Kilifi County referral hospital [18]. The malaria-positive fraction(s) applied in the computation are shown below.

**Table:** 

Year	1990	1996	2002	2008	2014	2020
Malaria positive fraction	0.4	0.48	0.50	0.12	0.22	0.22

Using the above information, we found that with a minimum of 37 cases, the study detected at least a 20% change in the annual incidence rates of endemic BL.

### Study procedures

Secondary anonymized data from participants meeting the inclusion criteria were utilized. In virtually all the instances, these data were retrieved from secure password protected electronic databases by the data manager at the KEMRI Center for Geographic Medicine Research Coast (CGMRC) (KEMRI-CGMRC). Permission for the same was granted by the KEMRI-CGMRC data governance committee.

The data were initially exported into Excel. These included final discharge diagnosis, available laboratory investigations, clinical history, pathology findings from biopsies, imaging, and treatment regimens used. Finally, the data were imported into STATA for manipulation and analysis.

### Data management and analysis

To ensure that no cases were missed, all available medical records for cases captured as pediatric cancers at admission discharge were initially reviewed by Michael Mwaniki and Shebe Mohammed. A second review was then conducted for those diagnosed with Burkitt’s lymphoma. For trend comparisons, data for all total pediatric admissions aged 0–14 years and total pediatric admissions with a final diagnosis at discharge of *P. falciparum malaria* were similarly prepared for manipulation and analysis. We conducted the final analysis via STATA (Stata Corp, College Station, TX, USA). Detailed analysis of the data set focused on demographic data, tumor site and presentation, and trend analysis of annual incidence rates controlled for the at-risk catchment population. Specifically, to test for the statistical significance of the incidence rates of Burkitt’s lymphoma across the three epochs, one-way ANOVA was performed. Similarly, one-way ANOVA was used to calculate the difference in malaria incidence across the three epochs, with the percentage positivity and malaria parasite density used as proxies. Finally, correction for the correlation between malaria and endemic Burkitt’s lymphoma was explored via linear regression, and the correlation coefficient was calculated.

## Results

The total number of admissions for the entire period from 1990 to 2020 was 124,298, of which 69,468 (56%) were males. Between 1990 and 1999, admissions increased by 236.7 per year (*p = < 0.0001*). In the second ten-year period, the number of admissions decreased by 129.2 for each unit change in year (*p* = 0.0029). The number of annual admissions then remained relatively unchanged during the last decade, showing only a marginal reduction of 8.6 admissions per year, a trend that was not statistically significant (*p* = 0.9105).

From the admission records, 95 patients with Burkitt’s lymphoma were identified, of whom 72 (75.8%) were male. The median age at diagnosis was 6.0 years, with an interquartile range (IQR) of 2.72–8.28 for boys and 7.0 (3.25–10.75) for girls, but the difference in age at presentation was not statistically significant (Mann‒Whitney U test 513.0, p value of 0.252). Most of the subjects presented with at least one primary mass in the jaw (67.7%). Many patients had secondary associated sites, especially the abdomen (40%), central nervous system (27%) and bone marrow (20%). Anemia (hemoglobin (Hb) < 10 g/dl) was found in 38 (48.7%) of the patients, whereas 10 (12.8%) of the patients had severe anemia, defined as Hb < 7 g/dl at admission. None of the patients had thrombocytopenia, defined as a platelet count less than 150,000 per microlitre. Furthermore, three subjects (4.6%) had jaundice, with four having clinically appreciable hepatomegaly and an additional 12 (12.6%) having splenomegaly. Twenty subjects had fever (> 37.5 °C). None of the patients were hypoxemic (pulse oximetry < 90%) on admission.

The annual hospital admission rates were calculated for endemic Burkitt’s lymphoma patients first, with the total number of pediatric admissions aged 0–14 years as the comparator. Furthermore, the same was recalculated via census estimates for the overall population at risk (0–14 years) in Kilifi County over the study period to determine the population incidence rate.

**Fig. 1:**
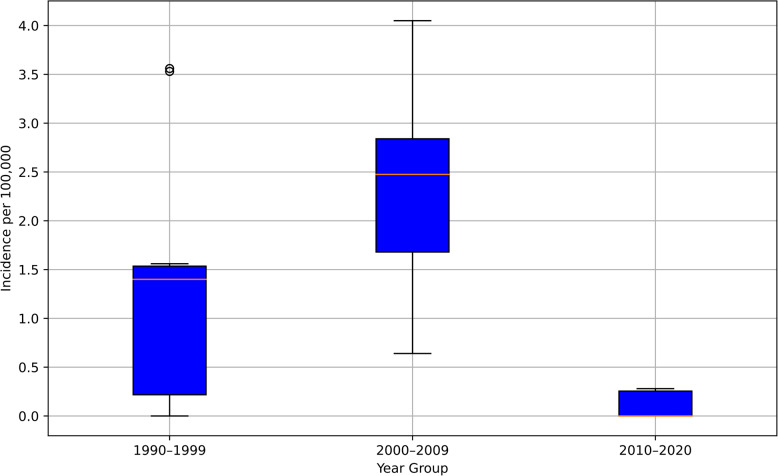
Incidence rate of endemic Burkitt’s lymphoma per 100,000 across the three epochs

The annual hospital admission incidence rates of Burkitt’s lymphoma increased continuously, with the highest admission incidence rate being 196.1 (95% CI 74.56–317.64) per 100,000 admissions in 2001. This corresponded to an incidence rate of 4.0 (95% CI 0.08–7.92) per 100,000 when the overall at-risk population in the catchment area was controlled (Table 1). The comparison between the 10-year periods revealed a decrease in new cases in the third epoch. The first period (1990–1999) had 37,589 admissions and 29 cases of Burkitt’s lymphoma, which translated to a 10-year cumulative incidence rate of 93.1 cases per 100,000. The second period had the highest number of cases of Burkitt’s disease (62 cases), translating to a 10-year cumulative incidence rate of 130.3 cases per 100,000. The final period had the lowest number of cases, at just 4, with a cumulative incidence of 10.2 cases per 100,000). When one-way ANOVA was used to test for statistical significance of the incidence rate of endemic Burkitt’s lymphoma across the three epochs, the F statistic within and between groups was significant (*p* < 0.0001) (Fig. 1), indicating that the overall decline was statistically significant.

**Table 1:** Incidence rates of Burkitt’s lymphoma per 100,000 admissions and per 100,000 under 14 population estimates

Year	Number of Burkitt’s Lymphoma cases admitted	Total Admissions	Incidence rate per 100,000 total admissions	95% CI	Incidence rate per 100,000 of the catchment population	95% CI
1990	0	2395	0.0	(0, 0)	0.0	0.00–0.00
1991	0	3549	0.0	(0, 0)	0.0	0.00–0.00
1992	0	2949	0.0	(0, 0)	0.0	0.00–0.00
1993	3	3127	95.9	(0, 204.44)	1.6	0.00-4.08
1994	7	4124	169.7	(43.97, 295.43)	3.5	0.00-7.17
1995	3	3747	80.1	(0, 170.72)	1.5	0.00-3.90
1996	3	4114	72.9	(0, 155.41)	1.4	0.00-3.72
1997	3	4305	69.7	(-0, 148.57)	1.4	0.00-3.72
1998	8	4182	191.3	(58.74, 323.86)	3.6	0.00-7.32
1999	2	5134	39.0	(0, 93.02)	0.9	0.00-2.76
2000	6	5126	117.1	(23.42, 210.78)	2.5	0.00-5.60
2001	10	5100	196.1	(74.56, 317.64)	4.0	0.08–7.92
2002	7	4797	145.9	(37.81, 253.99)	2.7	0.00-5.92
2003	6	5459	109.9	(21.96, 197.84)	2.3	0.00-5.27
2004	4	4963	80.6	(1.61, 159.59)	1.5	0.00-3.90
2005	8	4612	173.5	(53.28, 293.72)	2.9	0.00-6.24
2006	7	4819	145.3	(37.68, 252.92)	2.4	0.00-5.44
2007	10	4260	234.7	(89.22, 380.18)	3.4	0.00-7.01
2008	2	4010	49.9	(0, 119.04)	0.7	0.00-2.34
2009	2	4429	45.2	(0, 107.81)	0.6	0.00-2.12
2010	0	4035	0.0	(0, 0)	0.0	0.00–0.00
2011	0	3982	0.0	(0, 0)	0.0	0.00–0.00
2012	0	3367	0.0	(0, 0)	0.0	0.00–0.00
2013	0	2687	0.0	(0, 0)	0.0	0.00–0.00
2014	1	3932	25.4	(0, 75.22)	0.3	0.00-1.37
2015	1	4090	24.4	(0, 72.27)	0.3	0.00-1.37
2016	1	3660	27.3	(0, 80.83)	0.3	0.00-1.37
2017	0	2187	0.0	(0, 0)	0.0	0.00–0.00
2018	0	3752	0.0	(0, 0)	0.0	0.00–0.00
2019	0	4056	0.0	(0, 0)	0.0	0.00–0.00
2020	1	3327	30.1	(0, 89.05)	0.2	0.00-1.08

**Fig. 2:**
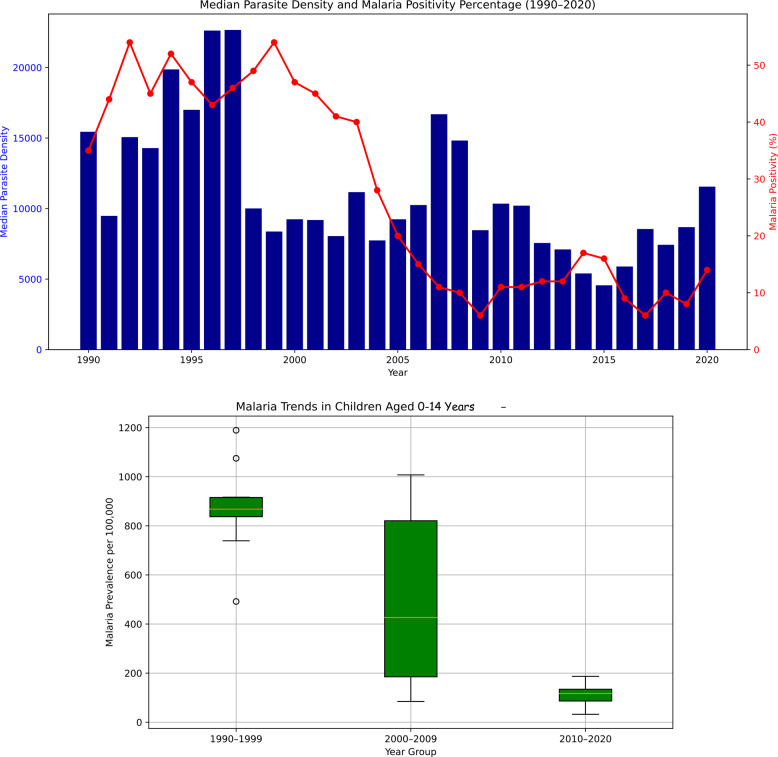
Trends of malaria parasite density and malaria positivity fraction and prevalence of malaria across the three epochs

In 1990, 847 cases were positive for *P. falciparum malaria*, out of 2,395 total admissions, resulting in a Falciparum malaria positivity percentage of 35% of all admissions. Overall, in the first epoch (1990–1999), the number of positive cases and the Falciparum malaria positivity fraction continued to increase, reaching their highest point in 1992, with 1,595 positive cases and a positivity percentage of 54%. The median parasite density in this first epoch was 13,966.5 (Q1:2090, Q3:126,000). In the second and third stages, the number of positive cases, total admissions, and positivity percentage decreased, with the lowest values occurring in 2017. The median parasite density also rapidly decreased over the two epochs, being 9,744 (Q1: 1560, Q3:114,000) and 7,224 (Q1:1566, Q3:109,200) in the second and third epochs, respectively. The decline across the three epochs was significant (Kruskal‒Wallis chi-square test, 12.3; *P* = 0.0021). This difference was similarly significant when one-way ANOVA was applied (*p* < 0.001) (Fig. 2).

**Fig. 3:**
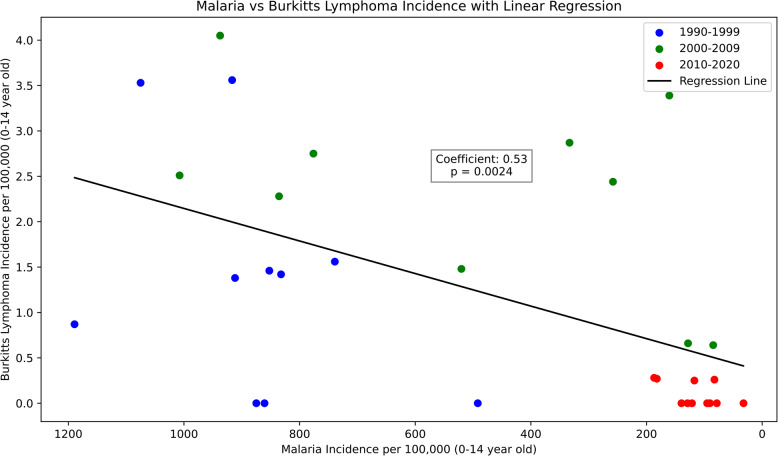
Incidence rate of endemic Burkitts lyphoma controlled for changing malaria period prevalence and for the at-risk population. Note: i) Each ten year epoch is represented with distinct identifying colour as shown in the key. ii) The annual incidence rate of endemic Burkitt’s lymphoma for every single year is marked accordingly across the three epochs. iii) The correlation coefficient between these two variables was approximately 0.53. This value indicates a strong positive correlation (*P*=0.0024), suggesting that as the malaria prevalence decreases, endemic Burkitt’s lymphoma incidence rate equally decreases.

Finally, to analyze the relationship between endemic Burkitt’s lymphoma cases and Falciparum malaria infection point prevalence, linear regression was applied, as displayed in Fig. 3. The correlation coefficient was 0.53, indicating a moderately strong positive correlation (*P* = 0.0024).

## Discussion

More than 100,000 children are diagnosed with cancer in the sub-Saharan Africa region [19]. These estimates are imprecise and grossly underestimate the burden largely because of the underdeveloped healthcare infrastructure across all aspects of cancer management (absent cancer registries, paucity of healthcare professionals trained in cancer diagnosis and management, poor access to treatment, and limited cancer research) in the region [19]. Overall, mortality is equally high, with a cure rate lower than 50% in many countries [6].

Non-Hodgkin lymphoma (NHL) is one of the most prevalent childhood cancers in sub-Saharan Africa [20]. The bulk (more than 50%) of NHLs are composed of BL, and the proportion of BL may exceed 60–80% within the endemic BL belt [21]. The endemic BL region generally overlies the Plasmodium malaria-endemic countries in tropical Africa [21]. Given the consistently documented overlay of the two conditions, repeated *P. falciparum malaria* infection has been postulated to play a central role in the pathogenesis of endemic BL, resulting in higher incidence rates [22]. However, this postulated association has never been fully examined. Therefore, this study is one of the largest and longest observational studies tracking both the incidence rates of endemic BL and *P. falciparum malaria* infections within the BL region.

Analysis of the cumulative BL cases over the study period revealed that the median age at presentation was approximately 6.0 years, with an IQR of approximately 3–8 years for boys and 7.0 (3–11) years for girls, which is similar to that reported in other studies [12]. The annual incidence rates were highest in the mid-1990s, at approximately 4.8 per 100,000 of the population at risk, which was within the 3–6 per 100,000 uniformly documented within the endemic Burkitt’s lymphoma belt [21]. From the mid-2000s, a gradual decline was recorded, reaching a nadir of 0.2 cases per 100,000 people by the year 2020.

Malaria infections were very common in the first 15 years (1990–2000 s), with close to 50% of all admitted children being diagnosed with falciparum malaria clinically as well as through a positive malaria blood smear. From the mid-2000s, the number of malaria-positive cases decreased to less than 20% of all pediatric admissions. This decline was sustained for the remainder of the study period. The year-over-year decline in the percentage of children admitted with malaria was significant (*P value = < 0.0001).* This significant decline in malaria infections has been documented in other hospitals and community-based studies [15, 18]. This phenomenon may be driven by a combination of preventive strategies, such as early effective treatment, indoor residue spraying, and the use of long-lasting insecticide-treated mosquito nets.

Malaria parasite density may correlate with population-level variation in infection burden [23], with high malaria prevalence areas tending to be associated with higher parasite density among those infected and vice versa [23]. In this study, the malaria parasite density closely followed a similar trajectory to that of the malaria-positive fractions. The year-over-year decrease in malaria parasite density across the study period was significant (*P value = < 0.0001).* Therefore, although this was a hospital-based study, a significant decrease in both the percentage of children admitted with positive malaria slides and malaria parasite density plausibly indicates an equally significant decline in overall *P. falciparum malaria* prevalence within the catchment population.

Finally, this study examined the correlation between *P. falciparum malaria* reduction and incidence rates of endemic BL. The correlation coefficient was 0.53, indicating a moderately strong positive correlation (*P* = 0.0024).

Since *P. falciparum malaria* is postulated to play a crucial role in the pathogenesis of endemic BL, reducing and/or eradicating malaria infection has been advocated as a plausible preventive strategy for endemic BL [24]. However, few studies have explored this relationship, and the uncertainties of any association still abound. Our study, which spans three decades, is one of the first to clearly demonstrate that a sustained reduction in *P. falciparum malaria* infection was associated with a significant decline in the incidence rates of endemic BL. Notably, a lag of at least half a decade was discernible between the years of noticeable reduction in the malaria positivity fraction and malaria parasite density before any reduction in annual cases of endemic BL was documented. This may imply that seasonal or short-lived variations in malaria incidence or transmission dynamics have negligible impacts on the incidence rates of endemic BL and that sustained reduction is a requirement. Furthermore, although the onset of partial immunity in older children leading to repeated cycles of infections that are cleared without treatment may play a role in the pathogenesis of the malignancy [10, 12], the time it takes for this to “*unlock*” the process leading to B-cell immortalization is unknown. It is therefore possible that the high number of endemic BL cases witnessed during the initial years of malaria infection reduction depicts individuals in whom the pathological process may have started in the earlier years of very high malaria prevalence.

Overall, our study demonstrates the transformation of an endemic BL picture into a level of annual incidence rates similar to those experienced within sporadic BL regions. Previous studies that have examined the relationships between BL and malaria have reported mixed findings. A study conducted in Uganda between 1976 and 2005 concluded that the distribution of Burkitt’s lymphoma did not differ across the three decades [25]. However, that study was conducted during decades of sustained high malaria prevalence in the region. Therefore, given the postulated role of *P. falciparum malaria* in this pathogenesis, it is unlikely that a discernible change would have been demonstrated. Another recently published article equally explored global trends in pediatric lymphomas [26]. That study noted an increase in BL across 8 of the 15 global regions. However, the study used data collected from 1988 to 2012, a period of high sustained malaria transmission [26]. In summary, the findings of our study are close to what was reported in a publication from Tanzania that utilized data collected between 2000 and 2009 [27]. That study revealed that the number of BL cases was greater in the first period (2000–2004) than in the second period (2005–2009), although there was uncertainty in the significance of the observed decline [27]. Notably, the Tanzania study utilized data across a much shorter observation period (one decade). Furthermore, this was conducted during a period of transition from the high malaria incidence and the initial years of declining malaria transmission, which may explain the observed uncertainty.

### Significance of the study findings

Pediatric cancers are expensive to diagnose and treat, with the estimated cost per diagnosis of a new single case being more than $31,000 [28]. With respect to endemic BL, treatment outcomes in sub-Saharan Africa remain suboptimal [29], and the cost for treating a single patient is estimated at close to $12,829 [30]. Although this puts the treatment costs within the boundaries of what would be considered cost effective by various models, including the WHO CHOICE model [30], it is still prohibitive as a direct cost within the fragile healthcare systems in most SSA countries. On the other hand, malaria control programs are among the most cost effective and can be deployed rapidly at scale [31]. The median financial cost of protecting one person for one year may be well under $5 for most of the proven interventions, such as insecticide-treated nets, indoor residue spraying, and intermittent preventive therapy, with a considerable benefit-to-cost ratio [31]. Therefore, our study demonstrates that malaria infection control programs such as the aforementioned (early effective treatment, indoor residue spraying, and the use of long-lasting insecticide-treated mosquito nets), may lead to a significant reduction in the burden of *P. falciparum malaria* infection as well as endemic BL, a malignancy that is responsible for 40–60% of the pediatric cancers within the endemic BL regions. This finding lends credence to the postulation of the association between BL and *P. falciparum malaria* as well as suggestions that sustained malaria prevention could be a viable control strategy for this malignancy.

### Limitations

First, the diagnostic capabilities for cancers in Kenya and other SSA regions [32] are suboptimal, which may result in under diagnosis. Second, there is a paucity of knowledge on community health-seeking behavior for childhood cancers. Myths and misconceptions on the prospects of treatment and outcomes may lead to many children not being brought to formal healthcare systems [33]. Third, this study was conducted at a single site in one country. The pattern of referral may have changed, although there was no increase in the number of BL cases in adjacent hospitals during this period. Fourth, although the results may be generalizable to other similar regions, multisite and multi-country studies are needed to address this important question equally.

## Conclusion

There has been a significant decrease in the annual incidence rate of endemic BL associated with a sustained reduction in malaria incidence. Importantly, this is the first study to effectively demonstrate such phenomena and hence lends credence to the postulated association between endemic BL and *P. falciparum malaria*. However, multisite, multi-country studies exploring this topic within the endemic BL belt are needed.

## Data Availability

Datasets generated and analyzed during this study are available upon direct request to the corresponding author via the Kenya Medical Research Institute.

## Abbreviations

ANOVA: Analysis of Variance
BL: Burkitt’s lymphoma
CGMRC: Centre for Geographic Medicine Research Coast
EBV: Epstein‒Barr virus
ERC: Ethical Review Committee
HB: Haemoglobin
IQR: Interquartile Range
KEMRI: Kenya Medical Research Institute
KNH: Kenyatta National Hospital
LMICS: Low- and Middle-Income Countries
MYC: Myelo-Cytomatosis gene
NHL: Non-Hodgkin lymphoma
UON: University of Nairobi
USA: United States of America
WHO: World Health Organization’s
